# Motives for the Use or Not of Protective Equipment for the Recreational Practice of Skiing and Snowboarding in Spanish Winter Stations

**DOI:** 10.3390/healthcare9121767

**Published:** 2021-12-20

**Authors:** Marcos Mecías-Calvo, Carlos Lago-Fuentes, Iker Muñoz-Pérez, Jon Mikel Picabea-Arburu, Álvaro Velarde-Sotres, Silvia Aparicio-Obregón, Rubén Navarro-Patón

**Affiliations:** 1 Facultad de Formación del Profesorado, Universidade de Santiago de Compostela, 27001 Lugo, Spain; marcos.mecias@usc.es (M.M.-C.); ruben.navarro.paton@usc.es (R.N.-P.); 2 Facultad de Ciencias de la Salud, Universidad Europea del Atlántico, 39011 Santander, Spain; carlos.lago@uneatlantico.es (C.L.-F.); jon.picabea@uneatlantico.es (J.M.P.-A.); alvaro.velarde@uneatlantico.es (Á.V.-S.); 3 Sport Training, RUNNEA, 48901 Barakaldo, Spain; 4 Facultad de Ciencias de la Salud, Universidad Isabel I, 09003 Burgos, Spain; 5 Physical Education and Sport Department, Faculty of Education and Sport, University of the Basque Country UPV/EHU, 01007 Vitoria-Gasteiz, Spain; 6 Departamento de Salud, Universidad Internacional Iberoamericana, Campeche 24560, Mexico; 7 Facultad de Ciencias Sociales y Humanidades, Universidad Europea del Atlántico, 39011 Santander, Spain; silvia.aparicio@uneatlantico.es

**Keywords:** sports protections, winter sports, recreative practice

## Abstract

The objective of this research was to analyze the motives for using or not using protections by recreational ski and snowboard athletes, specifically regarding gender and age in the winter resorts of Sierra Nevada and Alto Campoo (Spain). A total of 520 users participated in Sierra Nevada (*n* = 306 (58,8%)) and Alto Campoo (*n* = 214 (42.2%)); 257 of them were men (49.4%) and 263 (50.6%) were women; from 6 to 50 years old; classified by 4 stages of development (Childhood (*n* = 106 (20.4%); Teenagers (*n* = 110 (21.2%); Young adults (*n* = 101 (19.4%); Adults (*n* = 203 (39.0%)). For the data collection an ad hoc questionnaire was used (socio-demographic data, use/no use of protection, motives for the use). The data revealed that 76.5% used protections equipment, with the women being more likely to use protective equipment than men. Regarding age, young adults and adults were the ones using less protection. In relation to the motives of using protective equipment, security was the main motive for using it, while the reason to avoid using it was, most of the time, discomfort. Additionally, the childhood and teenager groups were the ones who reported, as motivation, family obligation, showing the importance of the influence of the parents.

## 1. Introduction

Snowsports are conducted at high speeds in environments with different natural and constructed risks [1]. Due to the exponential increase of ski and snowboard practitioners during the last 20 years, and with 400 million skiers or snowboarders around the world [2,3,4], a rise of the registered injuries associated to winter sports might be expected [5].

Most snow sport injuries occur due to falls [6]. Specifically, the most common regions injured are the knee for the skiers and wrist for the snowboarders [6]. Apart from these, some severe and particular injuries with chronic or catastrophic results, such as head injuries, might happen during practice [1].

Regarding the high risk of suffering an accident or injury, internal and external factors must be borne in mind when these two sports are practiced. The internal aspects are related to physiological and psychological variables, such as muscular strength or anxiety. The external factors include snowy weather conditions, the slope or the equipment used, and others [6,7]. Indeed, the impact of adverse internal and external factors on injury rates has been measured from 2.4 to 7 injuries/1000 activity days [8].

In order to reduce the number of traumatic injuries, the use of protective equipment, such as a helmet or spinal protector, has been previously recommended [5,9]. For instance, the use of helmet in winter sports can reduce the risk of head injuries from 15 to 60 % [10,11,12]. Additionally, the severity of these injuries is dependent on the protective equipment that the skier/snowboarder has used, the lack of use of a helmet being the reason for one of the most serious injuries [13,14]. However, more than 25% of skiers and snowboarders do not use it [15]. Likewise, a recent study conducted by Mecías-Calvo and colleagues [16] showed similar data (23.5%). Nevertheless, this study showed that the use of this protection was age and gender-dependent. That is, children and teenagers used more protective equipment than older ones, and women were more prone to use protections than men [16].

Several studies have reported the reduction of accidents thanks for the use of equipment or protections in snow sports among other athletic activities. For instance, the use of protective equipment reduced the number of injuries of upper and lower members during the last few years [5]. Concerning these protections, its use has increased exponentially during the last 30 years. For instance, the helmet sales increased 3.2 times during the 21th century in United States [1]. However, the use of equipment is not increasing at the same rate as the number of snows ports participants, as explained above.

Regarding the importance of the use of protections to reduce risk of accident and injuries, no previous studies have analyzed the motives to wear, or not to wear, this protective equipment when practicing winter sports. Knowing the relevance of sociodemographic factors, this study should be performed by every country. In this sense, Spain reported more than 5 million people practicing snow sports per year (https://www.atudem.es/, accessed on 29 October 2021). However, no previous studies have been performed analyzing these motives in Spanish population. For these reasons, the aim of this study was to analyze the motives for using protections by recreational ski and snowboard athletes (specifically regarding gender and age) in the winter resorts of Sierra Nevada and Alto Campoo (Spain).

## 2. Materials and Methods

### 2.1. Study Design

A cross-sectional descriptive study with winter sport practitioners was designed [17]. All participants were informed previously of the goals of the study and the further use of their data, explaining to them the privacy of their answers. The Investigational Review Committee of the Department of Physical Education and Sport Sciences of a Spanish university approved the research. The study was conducted in accordance with the Declaration of Helsinki and meets the European General Data Protection Regulation (EU GDPR; EU 2016/679).

### 2.2. Participants

A non-probabilistic selection of subjects from both winter stations (Sierra Nevada and Alto Campoo) was involved in this study [18], which freely decide to participate in this questionnaire. The inclusion criteria were: participants must be older than 6 years and younger than 64, split by development stage as follows: childhood (6 to 11 years old); teenagers (12 to 17 years old); young adults (18 to 24 years old); and adults (25 to 64 years old) [19]. As exclusion criteria, elite athletes were not included in the research.

### 2.3. Procedures

An ad-hoc questionnaire was designed by a group of experts, composed by experts in questionnaire researches (M.M and R.N) and researches with expertise in injury prevention (C.L. and I.M.). After different meetings among the members of this group, a first draft with twenty questions was designed to answer the main goal of this study. To confirm the content validity, a pilot study was performed in Alto Campoo winter station during November 2017 (*n* = 20; adults *n* = 7, young adults *n* = 5, teenagers *n* = 4, childhood *n* = 4). After this pilot test, no changes were needed on the questionnaire. This was finally composed by two main sections: socio-demographic data of the participants and questions related to the reasons to the use of protections in their snowsports practice. Consequently, the survey was provided via a printed sample in person before skiing between December 2017 and February 2018.

After giving their consent to participate in the study, the participants answered nine questions split into following sections: (a) socio-demographic data (four questions: age, gender, sport (ski or snowboard) and type of practice (recreational vs. competitive); and (b) questions related to the use of protections during practice and the reasons for wearing it or not (five questions).

### 2.4. Statistical Analysis

The registered variables were expressed through frequency tables in order to analyze the data. The association between variables was studied through the χ^2^ Pearson along with the Phi coefficient to compare the use of protection regarding the established variables (men vs. women) and the practiced sport (ski vs. snowboard). To study the development stage (childhood, teenagers, young adults, and adults) the Pearson χ^2^ was used along with the contingency coefficient. Additionally, the Pearson χ^2^ was displayed to find possible differences among the development stage, sex and the reasons to use/not use any protective equipment. Then, a subsequent correspondence analysis was set to show proximity relationship between variables.

The statistical analysis was carried out using the 24.0 version of the Statistical Package for the Social Sciences^®^ (SPSS), with a level of significance *p* < 0.05.

## 3. Results

A total of 520 recreative athletes, 306 from Sierra Nevada (58.9%) and 214 (41.1%) from Alto Campoo, participated in this research. A total of 257 were men (49.4%) and 263 (50.6%) women. Subjects were between 6 and 64 years old, classified according to 4 development stages (Childhood (*n* = 106 (20.4%); Teenagers (*n* = 110 (21.2%); Young adults (*n* = 101 (19.4%); Adults (*n* = 203 (39.0%)). Concerning the practiced sports (snowboard or ski), there are not significant differences for men (*p* = 0.066) nor for women (*p* = 0.563). That is, the use of protection does not depend on this variable.

Regarding the reasons to use some protection, the main motive to use them is security, followed by family obligation (Table 1). On the other hand, the discomfort during sport practice is the first reason not to use protections (Table 1).

Analyzing the results according to gender and regarding the motives to use or not protective equipment, significant differences were found between men and women (χ^2^ (4) = 45.8, *p* < 0.001; contingency coefficient = 0.284, *p* < 0.001). Women declare using protection due to security more than men (85.17% vs. 58.76%, respectively). At the same time, the main reason not to use any protective equipment amongst gender is the resulting discomfort during the practice (12.17% vs. 33.85% for women and men, respectively).

Concerning the relation between development stage and motives to use or not to use some protective equipment, differences were found among groups (χ^2^ (12) = 70.8, *p* < 0.001; contingency coefficient = 0.346, *p* < 0.001).

Furthermore, whether gender is taking into consideration, the significant differences between development stage groups are maintaining (χ^2^ (9) = 22.1, *p* = 0.009; contingency coefficient = 0.278, for women and χ^2^ (12) = 59.8, *p* < 0.001; contingency coefficient = 0.434 for men). In men, the main reason to wear protective equipment (the security) has remained fixed across development stages. However, the percentage of men who reported this motive decreased as age increased (Table 1). At the same time, the percentage of skiers and snowboarders that show the lack of using any protective equipment due to discomfort increases as the age of the group rises (Table 1). The childhood and teenager groups are the ones who report as motivation their family obligation (representing 5.7% and 8.2%, respectively). Additionally, women remain stable in their motivation for using protective equipment, independently of development stage group (Table 1). Unlike men, women use more sport protections (87.45 % vs. 65.37 % for women and men, respectively), and report a lower percentage of not use any protective equipment due to discomfort. Nevertheless, both genders reported an increase of discomfort (in older groups) as the main reason to not to use protections (Table 1).

**Figure 1:**
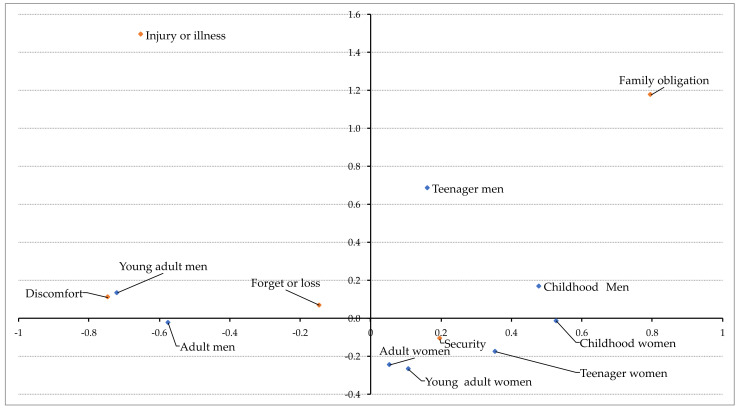
Correspondence analysis between stage development, sex, and reason to use/not use protective equipment.

Regarding possible differences between development stage, sex and reasons to use/not use protective equipment, Pearson χ^2^ test showed significant differences among variables (χ^2^ (28) = 142.54, *p* < 0.001; contingency coefficient = 0.262). The subsequent correspondence analysis (Figure 1) suggests an association between young adult men and the lack of use protective equipment for discomfort reasons. However, no further significant association was found.

## 4. Discussion

The aim of this study was to analyze the motives for using protections by recreational ski and snowboard athletes regarding gender and age in the winter resorts of Sierra Nevada and Alto Campoo (Spain).

Regarding the use of protections, only 76.5% of the participants claim to wear protections in snow sports, despite the benefit of using them to decrease risk of injuries [8,20] or minimizing their consequences [5]. This percentage is slightly higher than previous studies [2], which showed that 61.1% of Portuguese skiers and snowboarders used some protection during their practice. In fact, security was the main reason highlighted concerning the use of protection for both gender and different stage developments. Security is linked to the reduction of injury risk in several sports. For instance, the use of facial mask and helmet in ice hockey was included as mandatory in Canadian leagues to increase the security and reduce the risk of most common injuries, such as facial injuries and concussions (among others) [21].

However, the main reason to not use protection reported in our study was discomfort. Our findings match with previous studies, which indicated that users did not wear protections for their weight and rigidity, among other reasons [9]. Following this argument, a recent study suggests the need to design and adapt the protective equipment in snow sports to increase the comfort [22] as a strategy to help the use of protections in these sports. Indeed, a previous study reported that snowboarders who did not use helmet were more likely to sustain a head injury [23].

According to gender, the young adults group (from 18 to 24 years old) is the development stage with less protection use and so, the risk of an injury increases more than in other groups, being the men the ones who use less protections [24,25,26]. Women reported security as the main motive to wear protective equipment in their ski or snowboard practice (Figure 1), which it is especially significant in adult women, compared with men. This can be explained since men generally take more risks than women while practicing ski or snowboard [6,25,27], while women are usually more afraid in sports practice, decreasing the self-efficacy of the activity and thereby reducing its control [3].

Regarding the development stage, differences between young adults and adults (from 18 to 24 and from 25 to 64 years old) and childhood and teenagers (from 6 to 11 and from 12 to 17 years old) were found, the older ones being the ones using less protection (coinciding with previous studies) [25,27,28]. Analyzing the reasons to use protection, some relevant differences were reported according to the age. The family obligation was reported as the second main reason to use them in childhood and teenagers, meanwhile this reason was not reported in young adults and adulthood. The role of parents in the use of protection is crucial due to the low self-knowledge of security in these ages. Usually, children and young teenagers relate the use of protection, especially helmets, with something mandatory and not with a health protection [1,11]. The influence of the parents, regarding the use of helmets, is a key factor in order to change their mind. Therefore, it is recommended for the parents to insist and educate their children in the use of protection, even when it is not mandatory [25]. This finding matches with a previous study [29] which highlighted the necessity of encouragement from parents to use protection in snowboarding. However, this study also reported the requirement and friends as main factors or reasons to use protections. This knowledge should be used to design educational campaigns in this development stages [23].

Concerning young adults and adults, discomfort is more common and is associated with a lower frequency of the use of protections (Figure 1). This might be due to the fact that young adults and adults consider they have more ability to practice the sport, which generates a false sense of trust and, consequently, increases injury risk [30,31]. Nevertheless, adults above 60 years old should be taken into account, especially those whose physical condition can lead to more severe injuries compared to other groups of age [25]. The comfort should be one of the strategies to design appropriate protective equipment, especially for these development stages. However, more educative messages should be launched to increase the use of protection in this age group independently of discomfort. That is, discomfort protected users suffer a smaller number of injuries that comfort unprotected athletes. For these reasons, publicity campaigns and educational efforts may have led to a higher rate of helmet use in snowboarder and skaters [23].

Finally, some limitations need to be addressed. On the one side, the performance level of the participants was not asked, which could be a factor in deciding the use or not of protection [32], something which could be included in further studies. On the other side, results have to be taken carefully due to the design of study (cross-sectional and not longitudinal), due to the fact that nowadays there is no investigation regarding the evolution of the use of protection during the lifetime of a person. However, our novel findings will follow with furthers studies which gain in depth knowledge about other factors that can influence in the use of protective equipment in recreational snow sports practitioners, such as the influence of the different performance level (or hours of practice) or the existence of previous injury in these disciplines (related to the fear of recurrence). Other socioeconomic factors such as prices, lack of equipment, investment, and others should be investigated to analyze deeply other possible reasons regarding the use of protections in these sports. Lastly, attending the different reasons reported regarding the development stage, further studies should compare the discomfort reasons to analyze if the design, the weight, the rigidity, among others have the same relevance for each stage [9].

## 5. Conclusions

Despite the benefits of the protection during the practice of ski or snowboard, women use protection more than men, representing 76.5% of the total sample who use protections).

The most important factor towards using protection is security, and the reason to avoid its use is, mainly, discomfort. The influence of parents regarding the use of protection is essential to change this concept. It is recommended for parents to insist and educate their children in the use of protection, even when it is not mandatory. Despite skiing and snowboarding being considered safe sports, a proper awareness of using protective equipment, such as helmets or spinal protectors, with adequate corresponding behavior, could decrease the number of traumatic injuries. Furthermore, our results could help to manufacturers to improve the comfort of the protective material to increase the number of clients and, for sure, the security of sports practitioners.

## Figures and Tables

**Table 1:** Reasons why to use/not use protection regarding development stage and gender.

Stage	Childhood		Teenagers		Young Adults		Adults	
Use	Men	Women	Men	Women	Men	Women	Men	Women
Security	45 (42.5%)	46 (43.5%)	31 (28.2%)	51 (46.4%)	23 (22.8%)	42 (41.6%)	52 (25.6%)	85 (41.9%)
Family obligation	6 (5.7%)	4 (3.8%)	9 (8.2%)	2 (1.8%)	0 (0.0%)	0 (0.0%)	0 (0.0%)	0 (0.0%)
Injury or illness	0 (0.0%)	0 (0.0%)	1 (0.9%)	0 (0.0%)	1 (1.0%)	0 (0.0%)	0 (0.0%)	0 (0.0%)
**No use**								
Discomfort	3 (2.8%)	1 (0.9%)	12 (10.9%)	4 (3.6%)	26 (25.7%)	8 (7.9%)	46 (22.7%)	19 (9.4%)
Forget or loss	1 (0.9%)	0 (0.0%)	0 (0.0%)	0 (0.0%)	1 (1.0%)	0 (0.0%)	0 (0.0%)	1 (0.5%)

Data are presented as absolute and relative frequencies.

## Data Availability

The data presented in this study are available on request from the corresponding author.
